# Human Spermatogenesis: Insights From the Clinical Care of Men With Infertility

**DOI:** 10.3389/fendo.2022.889959

**Published:** 2022-05-18

**Authors:** Peter N. Schlegel

**Affiliations:** Department of Urology, Weill Cornell Medicine, New York, NY, United States

**Keywords:** human, spermatogenesis, male infertility, genetics, treatment

## Abstract

Human spermatogenesis is a qualitatively and quantitatively different process than that observed for most other mammals. In contrast with most other mammals, human spermatogenesis is characterized by reduced quantitative production and more abnormal sperm morphology. Until recently, direct evaluation and observations of human sperm production has been limited and the majority of scientific knowledge regarding spermatogenesis was derived from rodent models of study. Unique opportunities to observe human spermatogenesis have occurred as a consequence of the treatment of severe male infertility. These patients have sperm production so limited that no sperm reach the ejaculate so their fertility treatment involves surgical sperm retrieval from the testis, coupled with use of those sperm with advanced assisted reproductive techniques. Treatment of men with severe male infertility has enhanced identification of new genetic abnormalities that may cause this condition, since they now seek medical care. Three key novel concepts have resulted: (a) spermatogenesis is spatially heterogeneous in the human male, especially when sperm production is compromised, (b) genetic abnormalities are common in men with severe male infertility, particularly in men with diffuse maturation arrest and (c) rodent studies may not be an ideal model for understanding human male infertility. Scientific understanding of human spermatogenesis has been enhanced by these clinical observations.

## Introduction

With the advent of assisted reproductive technologies, the ability to treat severe forms of male infertility has been significantly enhanced. Spermatozoa that would have had limited chances of oocyte fertilization can now routinely be used for fertilization and subsequent pregnancy. In addition, the ability to make direct observations from use of single male gametes in assisted reproduction further increases our ability to understand the role of a male factor in human fertility.

The treatment of severe male factor infertility, where sperm production is so limited that no viable sperm are present in the ejaculate requires surgical sperm retrieval. This surgical intervention has also given us opportunities to directly investigate the internal function of the testis through clinical observation. The most effective form of sperm retrieval for non-obstructive azoospermia is microdissection testicular sperm extraction (microTESE) (1, 2) where direct evaluation of the seminiferous tubules within the testis is done to identify the sites of sperm production. In many cases, dissection is used to examine hundreds of the seminiferous tubules inside of the testis; a unique opportunity to evaluate and characterize spermatogenesis for these men. These observations provide the basis for some of the novel concepts described in this perspective manuscript.

Evaluation of men with severe male infertility now occurs routinely as part of their treatment process. The routine genetic testing for potential abnormalities such as Y chromosome microdeletions (3) prior to treatment has provided the opportunity to examine their genome on a broader scale than may otherwise have occurred. Our experience with treatment of men has resulted in a large number of men being referred for treatment, and, fortunately, most of them are willing to allow additional genetic testing under IRB oversight for novel conditions that may cause infertility, as well as the known causes of severe male infertility including karyotypic abnormalities and Y chromosome microdeletions (4).

## Heterogeneity of Human Spermatogenesis

Spermatogenesis in the rodent has been carefully characterized since the 1800s (5) and qualitatively documented since Clermont’s work in 1952 to have specific carefully coordinated and timed facets for the spermatogenic process (6). The documentation of 12 specific stages in the mouse reflects a uniquely organized process for spermatogenic development (7). Central to the observations of rodent spermatogenesis in nearly every publication is that the germ cell developmental process is uniform throughout the testis, with variations from tubule to tubule only based on tightly organized specific spermatogenic stages.

In distinction, human spermatogenesis has been typically characterized as being chaotic, with no reliable staging within the seminiferous tubule. Although attempts to define spermatogenic cycles in the human have been proposed by Nikkanen (8), and a complex interlocking spiral process of spermatogenic cell development was described by Schulze (9), human spermatogenesis is widely observed to be more chaotic than organized.

The treatment of men with non-obstructive azoospermia is complex since, by definition, these men have spermatogenesis so impaired that no sperm are observed in the ejaculate. As a result, surgery coupled with assisted reproduction is needed for fertility. Testicular histology is uniformly abnormal in these men. Interestingly, even men with predominant Sertoli cell-only pattern will have focal areas of sperm production identifiable with mTESE (10). Since sperm production is grossly abnormal in these testes, heterogeneity of sperm production is required for sperm production to be present in the testes; only men with at least focal spermatogenesis (different from their baseline or overall pattern of spermatogenesis) will have sperm retrieved. The microsurgical exploration and dissection of testicular tissue is needed to find these isolated areas of sperm production, reflecting why microTESE is the preferred method of sperm retrieval. Men with diffuse, uniform-appearing maturation arrest can be very challenging to treat. However, these men with diffuse maturation arrest will often have rare sperm in the ejaculate, even if wide dissection of their testicular tissue shows no obvious evidence of focally normal sperm production. So, even they have focal differences in sperm production despite a nearly uniform histologic appearance in the testes.

Large studies examining men with a normal karyotype have shown that only 52% of men will have normal spermatogenesis on testis biopsy, with the remainder showing globally decreased sperm production or late defects in spermatogenic development (11). Taken together, especially for men with impaired spermatogenesis, it is clear that human spermatogenesis is typically a heterogeneous process. This is markedly different from the process of spermatogenesis in other mammals, especially rodents.

## Genetic Abnormalities in Non-Obstructive Azoospermia

Defined genetic abnormalities causative of non-obstructive azoospermia are detected in approximately 20% of men with severely impaired sperm production, and include microdeletions of the AZFa, AZFb or AZFc regions of the Y chromosome as well as karyotypic abnormalities. Although Klinefelter syndrome is the more common karyotypic abnormality associated with non-obstructive azoospermia, autosomal translocations can also be found in men with this condition. Another 5% of men have a history of chemotherapy or radiation associated with their azoospermia, and about 10% of men have a history of cryptorchidism and prior orchiopexy. That leaves another 65% of men for whom we can either characterize their etiology based on histologic appearance (Sertoli cell-only and/or maturation arrest) or simply refer to them as being idiopathic. Since histologic characterization does not reflect an etiology, the majority of men with non-obstructive azoospermia could have a genetic cause for their severely impaired sperm production.

The recognition that infertility could be hereditary is not a new concept. In 1981, well before advanced genetic techniques identified specific genetic abnormalities, Cantu et al. recognized the presence of maturation arrest in 3 of 13 brothers from a consanguineous marriage, suggesting the possibility of an autosomal recessive genetic defect that could be causal for disordered spermatogenesis (12). In a limited population study, Fakhro et al. examined a cohort of 8 families using whole-exome sequencing to identify genes associating with non-obstructive azoospermia. They found that 10 of 16 men with infertility had novel genes with homozygous mutations segregating with the men who had infertility (but not present in their siblings). Of note, the majority of these five novel genes were associated with maturation arrest, with one associated with a Sertoli cell-only pattern. Gene expression was noted to be remarkably testis-specific, with evidence in experimental animals for their potential role in spermatogenesis for 4 of 5 genes. Among an additional 75 unrelated men, they found a 13% frequency of additional recessive variants, with no variants in fertile controls (13).

More recent evaluation of a population of 96 men from Northern Africa who were negative for karyotypic or Y microdeletion defects found 23% of these men had highly deleterious variants identified using a panel of only 151 genes. Six of the 16 variants identified in these 22 men had novel genes associated with their infertility (14). As discussed below, seven of the men had variants in piwi or DNA repair pathways with 12 having meiotic process gene defects identified. Of note, the men with defects in meiotic pathways did not have sperm retrieved, suggesting a potential prognostic role of such genetic testing.

Despite having a uniform, identifiable genetic abnormality, men may still result in a variable spermatogenic pattern within the testis. Deletions of AZFc are uniformly associated with impaired spermatogenesis; about 40% of these men are azoospermic and the remainder have severe oligospermia or even cryptozoospermia. However, within the testis, we commonly observe heterogeneity between different seminiferous tubules. So, although the AZFc deletion is the same in every cell of the body, individual tubules may have Sertoli cell-only, maturation arrest or hypospermatogenesis. Typically, each tubule will have the same pattern of spermatogenesis within the tubule, but an adjacent seminiferous tubule will often have a different histologic pattern. The explanation for such variation between tubules remains elusive, even when the genetic defect is uniform within the testis.

Unfortunately, genetic variants that cause spermatogenic failure may have unique or varied roles when evaluated in different ethnic groups or countries. For example, when Iberian investigators looked for 6 variants found in an Asian population, they observed that 3 variants were associated with spermatogenic failure in both additive and dominant models, with an associated negative predictive value for sperm retrieval for one of the variants. Of note, some of the variants are associated with lincRNAs, non-coding RNAs longer than 200 bp that are transcribed autonomously and do not overlap coding genes. It is widely accept that these lincRNAs control the expression of nearly genes in a tissue-specific manner. Of note, the testis represents the most enriched tissue in lincRNAs in humans (15).

The search for specific causal variants in populations with male infertility has been slow and tedious with only rare mutations identified using large study groups of well-characterized patients with severe male infertility. The genetic variants have also been identified as having a broad series of potential roles in spermatogenesis, including roles in genome integrity (16) as well as piRNA processing (16, 17). Definition of the specific cause of male infertility is particularly important, as it is now recognized that severe male infertility is a risk factor for future cancer development (4, 18). Testing that would allow clearer identification of the patient’s risk would be much more useful than simple counseling about “increased risk”. Patients are obviously confused and frustrated when increased risk exists but clinicians are unable to provide focused recommendations on how prior infertility patients should be screened for cancers. Specific identification of the causal etiology for infertility, whether a DNA repair defect or otherwise, would be critical for clinical recommendations in long-term follow up for cancer risk. Although accumulating evidence suggests that a genetic cause is common for severe male infertility, a clinically informative gene testing panel to aid in diagnosis is not currently widely available.

## Translation Between Rodent and Human Models

Rodents have remarkably high spermatogenic efficiency, and uniform patterns of histology, both of which are very different from human spermatogenesis. It is likely that a toxic effect on human fertility can occur without detection during screening in a rodent model. One example where rodent models have not been helpful is in the detection of the adverse effects of selective serotonin antagonists on male fertility potential. Whereas testosterone levels drop by 200 ng/dL and 50% of a cohort of normal men will have abnormal sperm DNA integrity produced within weeks of taking the SSRI (19), paroxetine, this defect was not detected in rodent models at high dose. This may be related to the fact that SSRIs act on sperm transport rather than sperm production, it is possible that the qualitatively and quantitatively limited human sperm production can be adversely affected by a drug, such as finasteride, without observing such an effect in a rodent model.

Another area where rodent models were limited in their ability to identify and/or quantify the role of specific genetic defects in spermatogenesis was for genes on the Y chromosome. In part, this relates to the observation that mammalian Y chromosomes can be highly divergent, but also that Y-gene targeting is made more difficult by the highly repetitive nature of the Y, also limiting genetic sequencing difficult with classical approaches (20). From a clinical standpoint, we know that several regions on Yq are critical for human spermatogenesis, including genes on AZFa as well as AZFb and SRY. However, it has been proposed by some that only two Y genes are essential for murine male fertility (21).

Certainly, there continue to be roles for murine models of spermatogenesis, to detect or confirm a putative genetic cause of male infertility. At a minimum, such data help to support statistical evaluations of an association between genetic variants and impaired spermatogenesis. However, there are substantial limitations to using a rodent model to predict human spermatogenesis.

## Summary

Human spermatogenesis is unique in mammalian models of testicular function. Not only should we avoid assuming that an observation in a rodent model will predict human testicular function, but continued work to evaluate human spermatogenesis directly will be required to understand male fertility.

## Data Availability Statement

The original contributions presented in the study are included in the article/supplementary material. Further inquiries can be directed to the corresponding author.

## Author Contributions

This work is based on the sole authors observations, writing, editing and review of final submission.

## Funding

The article was funded in part by NICHD P50HD096723, as well as The Reeve Foundation fund of Weill Cornell Medicine.

## Conflict of Interest

The author declares that the research was conducted in the absence of any commercial or financial relationships that could be construed as a potential conflict of interest.

## Publisher’s Note

All claims expressed in this article are solely those of the authors and do not necessarily represent those of their affiliated organizations, or those of the publisher, the editors and the reviewers. Any product that may be evaluated in this article, or claim that may be made by its manufacturer, is not guaranteed or endorsed by the publisher.

## References

[1] Schlegel PN. Testicular Sperm Extraction: Microdissection Improves Sperm Yield With Minimal Tissue Excision. Hum Reprod (1999) 14:131–5. doi: 10.1093/humrep/14.1.131 10374109

[2] Bernie AM Mata DA Ramasamy R Schlegel PN. Comparison of Microdissection Testicular Sperm Extraction, Conventional Testicular Sperm Extraction, and Testicular Sperm Aspiration for Nonobstructive Azoospermia: A Systematic Review and Meta-Analysis. Fertil Steril (2015) 104(5):1099–103. doi: 10.1016/j.fertnstert.2015.07.1136 26263080

[3] Girardi SK Mielnik A Schlegel PN. Submicroscopic Deletions of the Y Chromosome in Infertile Men. Hum Reprod (1997) 12:1635–41. doi: 10.1093/humrep/12.8.1635 9308784

[4] Schlegel PN Sigman M Collura B De Jonge CJ Eisenberg ML Lamb DL. Diagnosis and Treatment of Infertility in Men: AUA/ASRM Guideline Part I. J Urol (2021) 205:36–43. doi: 10.1097/JU.0000000000001521 33295257

[5] Niessing G. Investigations Into the Development and the Finest Structure of the Seminal Threads of Some Mammals. Treatises Phys Medic Würzburg Soc (1889) 22(2):35–63.

[6] Clermont Y Perey B. Quantitative Study of the Cell Population of the Seminiferous Tubules in Immature Rats. Am J Anat (1957) 100(2):241–67. 10.1002/aja.1001000205 13435229

[7] Oakberg EF. Duration of Spermatogenesis in the Mouse and Timing of Stages of the Cycle of the Seminiferous Epithelium. Am J Anat (1956) 99(3):507–16. doi: 10.1002/aja.1000990307 13402729

[8] Nikkanen V Söderström KO Parvinen M. Identification of the Spermatogenic Stages in Living Seminiferous Tubules of Man. J Reprod Fertil (1978) 53(2):255–7. doi: 10.1530/jrf.0.0530255 357716

[9] Schulze W. Evidence of a Wave of Spermatogenesis in Human Testis. Andrologia (1982) 14(2):200–7. doi: 10.1111/j.1439-0272.1982.tb03124.x 7103139

[10] Anniballo R Ubaldi F Cobellis L Sorrentino M Rienzi L Greco E. Tesarik Criteria Predicting the Absence of Spermatozoa in the Sertoli Cell-Only Syndrome can be Used to Improve Success Rates of Sperm Retrieval. J Hum Reprod (2000) 15(11):2269–77. doi: 10.1093/humrep/15.11.2269 11056118

[11] Chandley AC Maclean N Edmond P Fletcher J Watson GS. Cytogenetics and Infertility in Man. II. Testicular Histology and Meiosis. Ann Hum Genet (1976) 40(2):165–76. doi: 10.1111/j.1469-1809.1976.tb00176.x 1015811

[12] Cantú JM Rivas F Hernández-Jáuregui P Díaz M Cortés-Gallegos V Vaca G. Meiotic Arrest at First Spermatocyte Level: A New Inherited Infertility Disorder. Hum Genet (1981) 59(4):380. doi: 10.1007/BF00295476 6800930

[13] Fakhro KA Robay A Rodrigues-Flores JL Mezey JG Al-Shakaki AA Chidiac O. Point of Care Exome Sequencing Reveals Allelic and Phenotypic Heterogeneity Underlying Mendelian Disease in Qatar. Hum Mol Genet (2019) 28(23):3970–81. doi: 10.1093/hmg/ddz134 31625567

[14] Kherraf ZE Cazin C Bouker A Fourati Ben Mustapha S Hennebicq S Septier A. Whole-Exome Sequencing Improves the Diagnosis and Care of Men With Non-Obstructive Azoospermia. Am J Hum Genet (2022) 109:S0002–9297(22)00011-8. doi: 10.1016/j.ajhg.2022.01.011 PMC8948161 35172124

[15] Cerván-Martín M Bossini-Castillo L Rivera-Egea R Garrido N Luján S Romeu G. Effect and in Silico Characterization of Genetic Variants Associated With Severe Spermatogenic Disorders in a Large Iberian Cohort. Andrology (2021) 9(4):1151–65. doi: 10.1111/andr.13009 33784440

[16] Hardy JJ Wyrwoll MJ Mcfadden W Malcher A Rotte N Pollock NC. Variants in GCNA, X-Linked Germ-Cell Genome Integrity Gene, Identified in Men With Primary Spermatogenic Failure. Hum Genet (2021) 140:1169–82. doi: 10.1007/s00439-021-02287-y PMC8266742 33963445

[17] Nagirnaja L Mørup N Nielsen JE Stakaitis R Golubickaite I Oud MS. Variant PNLDC1, Defective piRNA Processing, and Azoospermia. N Engl J Med (2021) 385:707–19. doi: 10.1056/NEJMoa2028973 PMC7615015 34347949

[18] Eisenberg ML Betts P Herder D Lamb DJ Lipshultz LI. Increased Cancer Risk and Azoospermia. Fertil Steril (2013) 100(3):e12. doi: 10.1016/j.fertnstert.2013.06.025 23850299

[19] Tanrikut C Feldman AS Altemus M Paduch DA Schlegel PN. Adverse Effect of Paroxetine on Sperm. Fertil Steril (2010) 94:1021–6. doi: 10.1016/j.fertnstert.2009.04.039 19515367

[20] Subrini J Turner J. Y Chromosome Functions in Mammalian Spermatogenesis. Elife (2021) 10:e67345. doi: 10.7554/eLife.67345 34606444 PMC8489898

[21] Mazeyrat S Saut N Grigoriev V Mahadevaiah SK Ojarikre OA Rattigan A. A Y-Encoded Subunit of the Translation Initiation Factor Eif2 Is Essential for Mouse Spermatogenesis. Nat Genet (2001) 29:49–53. doi: 10.1038/ng717 11528390

